# HOTAIR/miR-1277-5p/ZEB1 axis mediates hypoxia-induced oxaliplatin resistance via regulating epithelial-mesenchymal transition in colorectal cancer

**DOI:** 10.1038/s41420-022-01096-0

**Published:** 2022-07-07

**Authors:** Xingyue Weng, Hao Liu, Jian Ruan, Miaoyan Du, Lingjie Wang, Jiayan Mao, Ying Cai, Xuemei Lu, Wei Chen, Yaqing Huang, Xiao Zhi, Jianzhen Shan

**Affiliations:** 1 Department of Medical Oncology, The First Affiliated Hospital, Zhejiang University School of Medicine, Hangzhou, People’s Republic of China; 2 Cancer Institute of Integrated Traditional Chinese and Western Medicine, Zhejiang Academy of Traditional Chinese Medicine, Tongde Hospital of Zhejiang Province, Hangzhou, 310012 Zhejiang China; 3 Reproductive Medicine Center, Department of Gynecology and Obstetrics, Zhejiang Provincial Peoples Hospital, Affiliated Peoples Hospital, Hangzhou Medical College, Hangzhou, 310014 Zhejiang China; 4 Department of Hepatobiliary and Pancreatic Surgery, the First Affiliated Hospital, Zhejiang University School of Medicine, Hangzhou, People’s Republic of China

**Keywords:** Cancer, Cell biology

## Abstract

The hypoxic microenvironment contributes to the chemoresistance of many malignant tumors including colorectal cancer (CRC). Accumulating studies have indicated that long non-coding RNAs (lncRNAs) play important roles in chemotherapy resistance. In this study, we aimed to determine the effect of lncRNAs in hypoxia-mediated resistance in CRC and its potential mechanism. Here, we discovered that hypoxia-induced oxaliplatin resistance and HOX transcript antisense RNA (HOTAIR) expression was increased in hypoxia-treated CRC cell lines and CRC tumors. Knockdown of HOTAIR by siRNA reduced the viability and proliferation of CRC cells treated with oxaliplatin and reversed hypoxia-induced resistance. Mechanically, we found that HOTAIR modulates zinc finger E-box binding homeobox 1 (ZEB1) expression by negative regulations of miR-1277-5p. When miR-1277-5p was silenced, knockdown of HOTAIR was unable to reduce the oxaliplatin resistance in CRC cells. In mouse models of CRC, HOTAIR knockdown markedly inhibited the tumor growth when treated with oxaliplatin. Thus, HOTAIR/miR-1277-5p/ZEB1 axis appears a promising therapeutic target for improving the oxaliplatin efficacy in CRC.

## Introduction

Colorectal cancer (CRC) is a common malignant tumor with a high rate of cancer-related mortality, which causes a significant medical burden worldwide [1]. According to the data from Cancer Statistics in China from 2015 [2], the incidence of CRC is increasing due to the accelerated growth in the elderly population. Fortunately, advances in strategies for early diagnosis and perioperative management have significantly improved long-term tumor-free survival [3]. However, similar to most solitary malignancies, post-operative tumor recurrence and metastasis are the major limiting factors for a better prognosis of CRC. Previously, the American Cancer Society reported that approximately 20% of CRC patients not only presented metastasis at diagnosis but also suffered a high rate of tumor relapse after surgery [4]. Adjuvant chemotherapy is another optimal treatment option. Administering fluoropyrimidine monotherapy could reduce the risk of tumor recurrence and mortality by 20–30% [5] and its combination with oxaliplatin promoted additional improvements in the tumor-free survival rate [6]. However, patients often acquire oxaliplatin resistance after its frequent and chronic oxaliplatin treatment, therefore there is an urgent need to investigate the underlying molecular mechanism of chemoresistance in CRC.

The mechanisms of drug resistance are varied, among which, spontaneous genetic modifications are the cause of de novo drug resistance, while changes in the tumor microenvironment (TME) are mostly related to acquired drug resistance [7]. As the increased proliferation of tumor cells leads to a hypoxic microenvironment within the tumor core, tumor cells adapt to altered TME and hypoxia conditions through various signaling pathways, promoting cancer progression and acquired drug resistance [8]. Therefore, the hypoxic microenvironment is considered to be a key factor causing tumor drug resistance.

Long non-coding RNAs (LncRNAs) comprise RNAs of ~200 bp to 100 kb, which represent a large percentage of the RNA transcribed from mammalian genomes. LncRNAs have become new major regulators in the initiation and development of multiple malignancies [9]. In addition, lncRNAs affect the response to chemotherapy by regulating various signaling pathways [10, 11]. Recently, a group of lncRNAs such as CRNDE, MALAT1, and ANRIL was identified as critical regulators of oxaliplatin resistance [12]. Therefore, seeking differential lncRNAs under hypoxia conditions may be a promising approach to reverse hypoxia-induced oxaliplatin resistance.

LncRNA HOX transcript antisense RNA (HOTAIR) was first discovered to promote invasion and metastasis in breast cancer cells via interacting with polycomb repressive complex 2 (PRC2) [13]. Recently, the critical role of HOTAIR in cancer development has been well investigated and a large body of evidence demonstrates that HOTAIR is elevated in cancer and correlates with metastasis and poor prognosis [14–18]. Although these studies disclosed the function of *HOTAIR* in cancer, the effect of *HOTAIR* on hypoxia-induced chemoresistance remains poorly understood.

In this study, we confirmed hypoxia-induced oxaliplatin resistance. We compared the lncRNA expressions in normal and hypoxia conditions and selected HOTAIR lncRNA. We verified that knockdown of HOTAIR partially reversed hypoxia-induced oxaliplatin resistance and the accompanied epithelial-mesenchymal transition (EMT) by the regulation of miR-1277-5p targeting zinc finger E-box binding homeobox 1(ZEB1).

## Results

### Hypoxia-induced oxaliplatin chemoresistance and enhanced HOTAIR expression in CRC cells

**Fig. 1:**
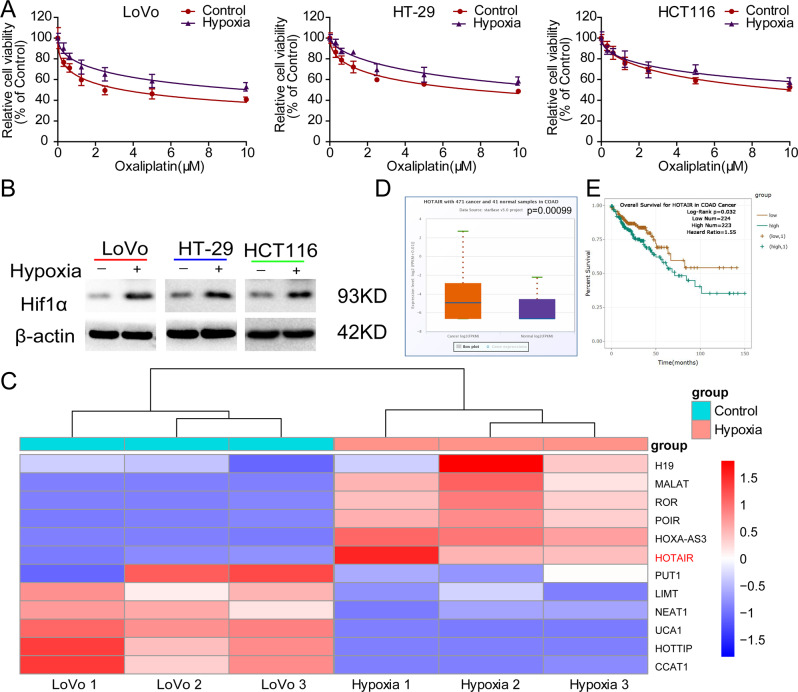
Hypoxia-induced oxaliplatin chemoresistance and enhanced HOTAIR expression in CRC. **A** Dose-response inhibition of CRC cells (LoVo, HT-29, and HCT116) to oxaliplatin under normoxia or hypoxia conditions. The relative cell viability of CRC cells was detected by CCK-8 assay. **B** Comparison of HIF-1α protein level in CRC cells between hypoxia and normal treatments. **C** Changes in lncRNA expressions under hypoxic treatment in LoVo cells. **D** Comparison of HOTAIR expression in CRC tissues to normal tissues. **E** Kaplan–Meier survival curve of COAD cancer patients with different HOTAIR expression (high vs low).

Previous work found that CRC cells such as HCT116 cells displayed enhanced resistance to oxaliplatin under hypoxic conditions [19]. To further clarify the roles of hypoxia in oxaliplatin resistance in CRC, we examined the cell viability of three different CRC cell lines (LoVo, HT-29, and HCT116) treated with oxaliplatin for 48 h under normoxia and hypoxia conditions. The dose-response curves showed that the viability of all three cell lines decreased with increases in oxaliplatin concentration, and hypoxia suppressed the relative cell viabilities of CRC cells compared with the group under normoxia (Fig. 1A), confirming that hypoxia increases the oxaliplatin resistance in CRC cells. The expression of hypoxia-inducible factor 1 subunit alpha (HIF-1α) was increased in all three cell lines with hypoxic treatment (Fig. 1B). Next, we sought to identify the lncRNA that can be involved in hypoxia-mediated oxaliplatin resistance in CRC. By analyzing some lncRNA expressions, we found that HOTAIR, HOXA-AS3, POIR, ROR, and MALAT were significantly upregulated in LoVo cells treated with hypoxia compared with the normoxia group (Fig. 1C). We were interested in HOTAIR. In addition, we analyzed the data from starBase v3.0 (http://starbase.sysu.edu.cn/) and confirmed that the expressional level of HOTAIR was significantly increased in tumor tissues compared with that in adjacent normal tissues (Fig. 1D). Moreover, survival analysis confirmed that patients with high HOTAIR expression presented a poor prognosis for CRC (Fig. 1E). These data suggest that HOTAIR might be involved in the hypoxia-enhanced oxaliplatin chemoresistance and hence poor prognosis.

### Knockdown of HOTAIR sensitized CRC cells to oxaliplatin

**Fig. 2:**
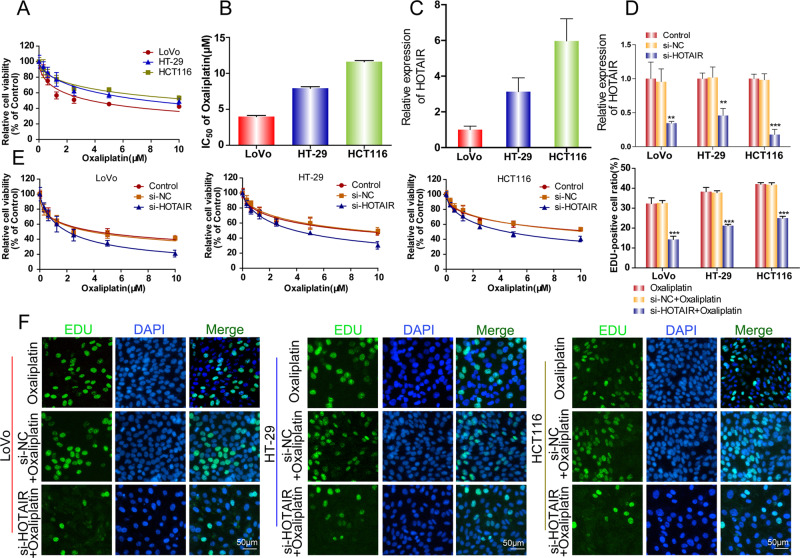
Deletion of HOTAIR sensitized CRC cells to oxaliplatin. **A**, **B** The relative cell viability of CRC cells was detected by CCK-8 assay and the value of IC50 was shown. **C** *HOTAIR* expression level was detected in CRC cells by qRT-PCR. **D** qRT-PCR was used to detect the expression of HOTAIR after si-HOTAIR treatment. **E** CCK-8 was used to detect cell viability after si-HOTAIR treatment and exposure to oxaliplatin. **F** EdU was used to detect cell proliferation after si-HOTAIR treatment and exposure to oxaliplatin. *p < 0.05, **p < 0.01.

To further explore the roles of HOTAIR in oxaliplatin resistance, we first compared the sensitivity of three CRC cell lines (LoVo, HT29, and HCT116) to oxaliplatin. The dose-response curve of oxaliplatin showed that LoVo cells were the most sensitive to oxaliplatin and HCT116 cells had the strongest oxaliplatin resistance (Fig. 2A). The half-maximal inhibitory concentration (IC50) values of oxaliplatin for HCT116 cells were almost threefold of IC50 for LoVo cells (Fig. 2B). Interestingly, the expression level of HOTAIR was highest in HCT116 cells, moderate in HT29 cells, and lowest in LoVo cells, indicating that HOTAIR expression correlates positively with oxaliplatin resistance (Fig. 2C). We then performed siRNA transfection to reduce the expression of HOTAIR in all three CRC cell lines (Fig. 2D). After the knockdown of HOTAIR, the viability of the three CRC cell lines was significantly decreased in the presence of oxaliplatin (Fig. 2E). Additionally, EdU staining verified that HOTAIR knockdown suppressed cell proliferation in CRC cells treated with oxaliplatin (Fig. 2F).

**Fig. 3:**
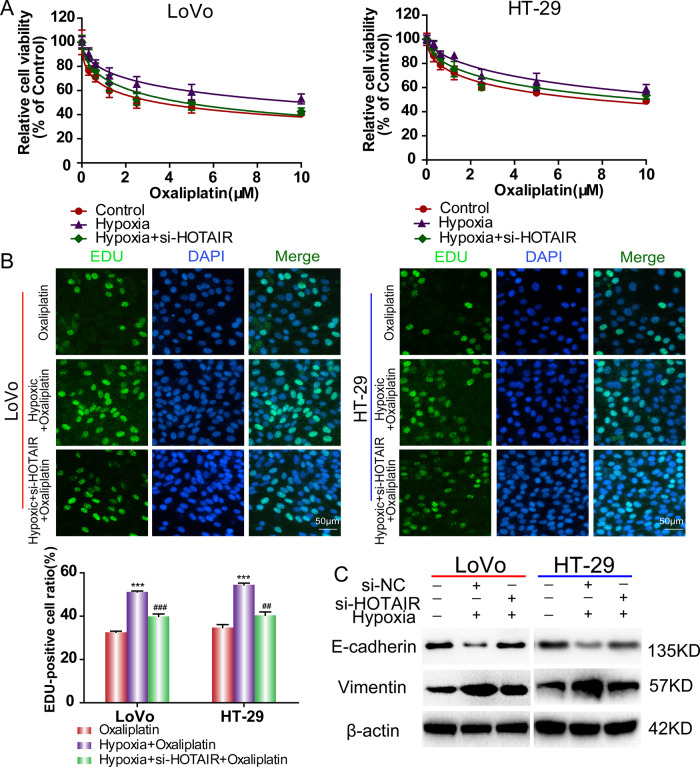
HOTAIR silencing restored the cytotoxicity of oxaliplatin in CRC cells under hypoxia. **A** CCK-8 was used to detect cell viability after si-HOTAIR treatment and exposure to oxaliplatin under hypoxic treatment. **B** EdU was used to detect cell proliferation after si-HOTAIR treatment and exposure to oxaliplatin under hypoxic treatment. **C** The western blotting assay was used to detect the E-cadherin and Vimentin protein levels under hypoxic treatment.***p < 0.001. ^##^*p* < 0.01, ^###^*p* < 0.001.

Given that hypoxia enhances oxaliplatin resistance in CRC cells (Fig. 1), we then explored the effect of silencing of HOTAIR on hypoxia-induced resistance. We transfected HOTAIR siRNA into CRC cells (LoVo cells and HT-29 cells) following exposure to hypoxia. Compared with the control group treated with hypoxia only, cells transfected with si-HOTAIR greatly reversed the hypoxia-induced increase in cell viability (Fig. 3A). In addition, HOTAIR knockdown by si-HOTAIR also significantly reduced hypoxia-induced cell proliferation when CRC cells were treated with IC50 concentration of oxaliplatin for 48 h (Fig. 3B). The results demonstrated that increased HOTAIR expression under hypoxia confers oxaliplatin resistance in CRC.

### HOTAIR enhanced the oxaliplatin resistance via activation of EMT in CRC cells

Emerging evidence indicates that there is a link between EMT and oxaliplatin resistance in cancers [20]; therefore, we measured EMT marker expression in CRC cells under oxaliplatin treatment. The results of confocal microscopy and western blotting confirmed that oxaliplatin-induced upregulated Vimentin expression and downregulated E-cadherin expression, which are hallmarks of EMT (Fig. S1). However, siRNA-mediated knockdown of HOTAIR partly reversed this process (Fig. S1). Next, we measured the changes in expression of E-cadherin and Vimentin in LoVo and HT29 cells treated with 1% O2 for 48 h. Such hypoxic treatment decreased E-cadherin expression and increased Vimentin expression in LoVo and HT29 cells, whereas knockdown of HOTAIR reversed the changes in EMT marker proteins induced by hypoxia (Fig. 3C). All of the above results indicated that HOTAIR is involved in oxaliplatin and hypoxia-mediated EMT signaling pathway.

### HOTAIR regulated EMT in a ZEB1-dependent manner

**Fig. 4:**
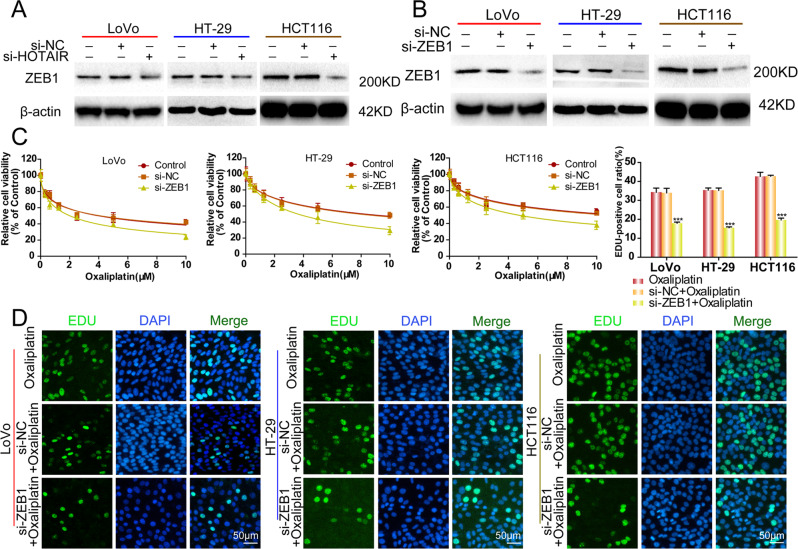
HOTAIR’s effects on CRC cells are associated with ZEB1. **A**, **B** The western blotting assay was used to detect the ZEB1 expression after si-HOTAIR treatment or si-ZEB1 treatment. **C** CCK-8 was used to detect cell viability after si-ZEB1 treatment and exposure to oxaliplatin. **D** EdU was used to detect cell proliferation after si-ZEB1 treatment and exposure to oxaliplatin. ***p < 0.001.

Previous reports revealed that Zinc finger E-BOX binding homeobox 1 (ZEB1) activates EMT [21, 22]. In the present study, we explored whether ZEB1 connects HOTAIR and EMT during the oxaliplatin resistance in CRC cells. We found that ZEB1 protein levels were suppressed in all three CRC cell lines transfected with si-HOTAIR (Fig. 4A). Next, we explored the effects of ZEB1 expression on chemosensitivity and EMT in CRC cells. *ZEB1* knockdown CRC cells were established using siRNA-ZEB1 transfection (Fig. 4B). Knockdown of ZEB1 significantly increased the sensitivity of CRC cells to oxaliplatin in all three CRC cell lines (Fig. 4C). Additionally, the cancer cell proliferation was markedly suppressed by si-ZEB1 (Fig. 4D). Moreover, ZEB1 knockdown reversed the change in EMT markers induced by oxaliplatin treatment (Fig. S2).

Furthermore, we compared the oxaliplatin sensitivity between CRC cells transfected with siRNA-ZEB1 and cells transfected with both siRNA-ZEB1 and siRNA-HOTAIR. The results showed that there was no significant difference in cell viability and the expression of E-cadherin and Vimentin between the siRNA-ZEB1 group and the siRNA-ZEB1 plus siRNA-HOTAIR group (Fig. S3). These results clarified that HOTAIR promoted EMT to increase CRC cell oxaliplatin resistance in a ZEB1-dependent manner.

### MiR-1277-5p is involved in the signaling transduction from HOTAIR to ZEB1

**Fig. 5:**
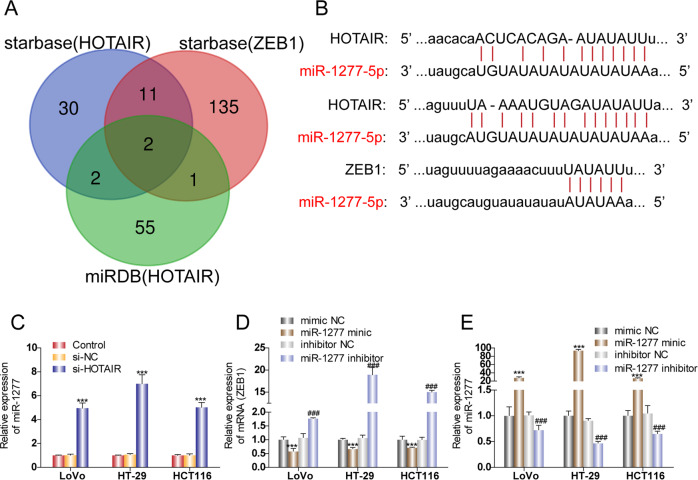
MiR-1277-5p is involved in the cross-talk between HOTAIR and ZEB1. **A** Veen diagram of candidate miRNAs. **B** Schematic illustration of the predicted miRNA (miR-1277-5p/miR-326) binding sites in HOTAIR and ZEB1 3'-UTR. **C** The relative expression level of miR-1277-5p after knocking down HOTAIR. **D** The relative expression level of ZEB1 after overexpressing miR-1277-5p or inhibiting miR-1277-5p. **E** The relative expression level of miR-1277-5p was confirmed after transfection of miR-1277 mimic, miR-1277-5p inhibitor, or corresponding negative control, respectively. ***p < 0.001, ^###^*p* < 0.001.

Because HOTAIR lacks the RNA sequences complementary to ZEB1 mRNA, we performed a screen for miRNAs that could potentially interact with both HOTAIR and ZEB1 mRNA, thus mediating the connection between HOTAIR and ZEB1. As shown in Fig. 5A, starBase and miRDB databases were utilized to predict the downstream target miRNAs of HOTAIR. Four miRNAs were identified as the predicted common miRNAs from both the starBase database (45 miRNAs) and the miRDB database (60 miRNAs). We then screened the starBase database and found that 149 miRNAs can bind ZEB1, among which two miRNAs (miR-1277-5p and miR-326) were determined to bind both HOTAIR and ZEB1 in all three screens (Fig. 5A). We chose to explore the function of miR-1277-5p. The software predicts the binding sites of HOTAIR with miR-1277-5p and ZEB1 with miR-1277-5p (Fig. 5B). To examine whether HOTAIR functions as ceRNA, CRC cells were transfected with HOTAIR siRNA. MiR-1277-5p expression was significantly increased by HOTAIR silencing in the three CRC cell lines (Fig. 5C), indicating that HOTAIR expression downregulates miR-1277-5p expression. We then altered the expression of miR-1277-5p by miR-1277-5p mimic and miR-1277-5p inhibitor and tested how miR-1277-5p expression level affects ZEB1 expression. The relative expression level of miR-1277-5p was confirmed in three CRC cell lines after transfection of miR-1277-5p mimic, miR-1277-5p inhibitor, or corresponding negative control, respectively (Fig. 5E). ZEB1 expression was much lower in CRC cells transfected with miR-1277-5p mimic compared to that in cells transfected with negative control, whereas ZEB1 expression was higher in CRC cells transfected with miR-1277-5p inhibitor than the corresponding NC group (Fig. 5D). The relation of HOTAIR, miR-1277-5p, and ZEB1 under hypoxia was also confirmed (Fig. S4). Hypoxia induced increase of HOTAIR and ZEB1 and decrease of miR-1277-5p. The results demonstrated that miR-1277-5p acts downstream of HOTHAIR to provide negative regulation of ZEB1.

### Knockdown of HOTAIR sensitized CRC cells to oxaliplatin by regulating miR-1277-5p

**Fig. 6:**
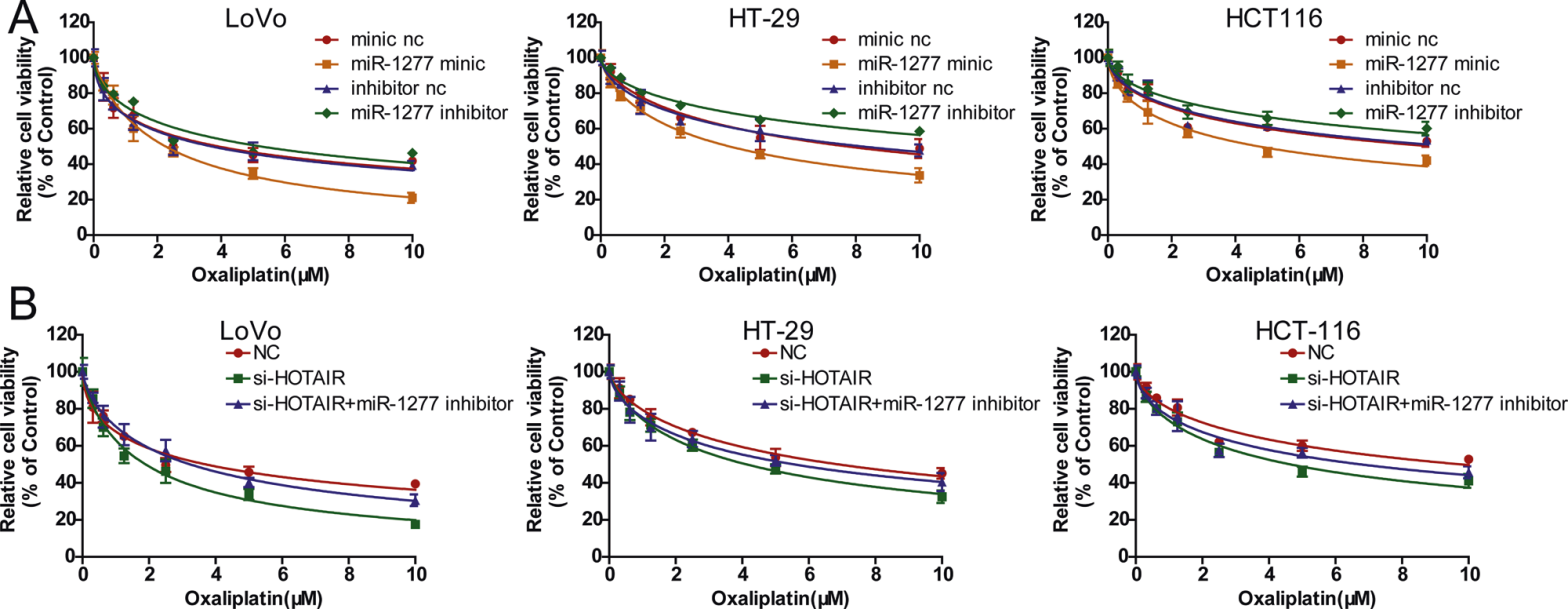
Knockdown of HOTAIR sensitized CRC cells to oxaliplatin by regulating miR-1277-5p. **A** CCK-8 was used to measure the cell viability of CRC cells transfected with miR-1277-5p mimic, miR-1277-5p inhibitor, or corresponding negative control. **B** CCK-8 was used to measure the cell viability of CRC cells transfected with NC, HOTAIR siRNA, HOTAIR siRNA+ miR-1277-5p inhibitor.

We next investigated whether miR-1277-5p is involved in the HOTAIR-mediated oxaliplatin resistance by changing the expression levels of miR-1277-5p in CRC cells with miR-1277-5p mimic or miR-1277-5p inhibitor. Upregulation of miR-1277-5p increased the sensitivity of oxaliplatin, while downregulation of miR-1277-5p decreased oxaliplatin sensitivity (Fig. 6A). Additionally, the cell viability of CRC cells transfected with HOTAIR siRNA was reduced with the treatment of oxaliplatin, while the cell viability of CRC cells co-transfected with HOTAIR siRNA and miR-1277-5p inhibitor was partially recovered (Fig. 6B), indicating that HOTAIR promotes oxaliplatin resistance via the negative regulation of miR-1277-5p.

### HOTAIR knockdown improved the effect of oxaliplatin therapy in vivo

**Fig. 7:**
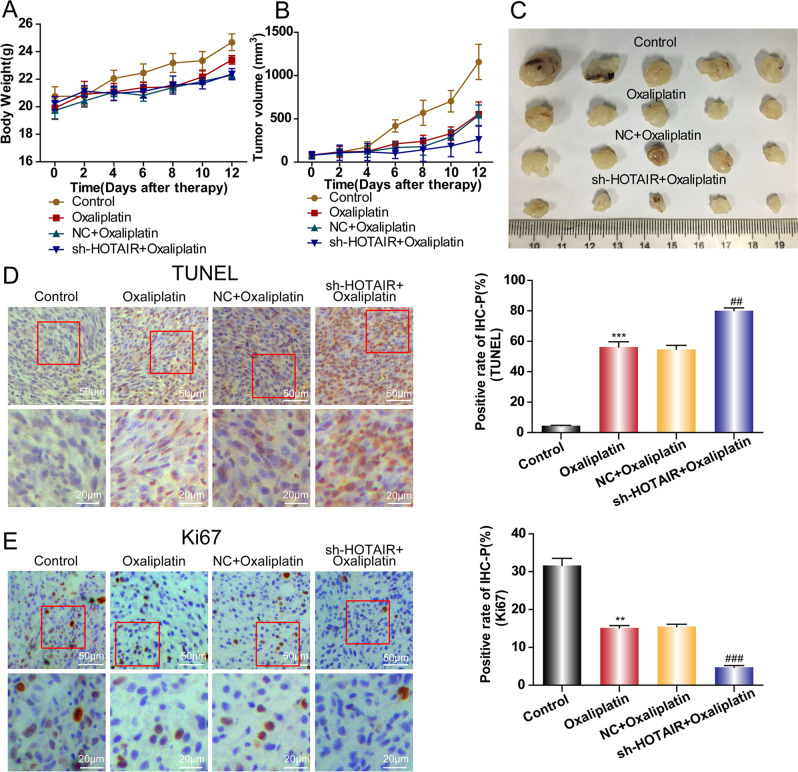
In vivo effects of HOTAIR knockdown. The tumor weight (**A**) and volume (**B**) in each group were statistically analyzed. **C** Representative images of xenograft tumors. **D** The cell apoptosis in each group was detected by a TUNEL assay. **E** The Ki-67 expression in each group was detected by Immunohistochemistry assay. **p < 0.01,***p < 0.001, ^##^*p* < 0.01, ^###^*p* < 0.001.

To further investigate whether HOTAIR is the potential therapeutic target for the treatment of CRC in clinical practice, a combination of sh-HOTAIR lentivirus with oxaliplatin therapy was delivered to the CRC mice models in vivo. Consistent with the results in vitro, HOTAIR knockdown along with oxaliplatin treatment decreased the tumor weight and volume compared with that in the control group treated with oxaliplatin only (Fig. 7A–C). Moreover, the terminal deoxynucleotidyl transferase nick-end-labeling (TUNEL) assay showed that the apoptosis rate was significantly higher in the sh-HOTAIR plus oxaliplatin treatment group than that in the oxaliplatin alone group (Fig. 7D). Conversely, the percent of Ki-67 positive cells was lowest in tumors with sh-HOTAIR plus oxaliplatin therapy (Fig. 7E), indicating the tumor cell proliferation was largely arrested by the treatment of sh-HOTAIR and oxaliplatin. These in vivo results confirmed that HOTAIR knockdown increased oxaliplatin sensitivity in CRC.

## Discussion

CRC is a prevalent and highly aggressive malignant tumor. Most patients with CRC initially appeared to respond well to the chemotherapeutic agent oxaliplatin; however, tumor resistance remains a major cause of therapy failure. Therefore, it is of great significance to explore the mechanism of drug resistance to improve the efficacy of chemotherapy. It has now become widely accepted in recent years that the dysregulation of lncRNAs is involved in cancer in different stages, including cancer initiation, progression, and chemoresistance. It is well known that the rapid proliferation of solid tumor cells results in intratumoral hypoxia, which profoundly affects cell proliferation, metastasis, and the cell response to chemotherapeutics [23]. However, studies regarding whether lncRNAs contribute to hypoxia-mediated resistance are very limited. Lin et al. reported that hypoxia-induced lncRNA LUCAT1 facilitates the growth of CRC cells and contributed to drug resistance of CRC cells both in vitro and in vivo [24]. Zhu et al. reported that lncRNA-EMS/miR-758-3p/WTAP axis regulates hypoxia-mediated drug resistance to cisplatin in esophageal cancer [25]. In the current study, we screened potential lncRNAs that may be crucial for hypoxia-induced oxaliplatin resistance in CRC. We discovered that lncRNA HOTAIR was significantly upregulated in hypoxia conditions compared with that in normal conditions. HOTAIR knockdown could partially reverse hypoxia-induced oxaliplatin resistance. Mechanically, HOTAIR functions as a ceRNA by sponging miRNA. These findings suggest that HOTAIR might act as a useful biomarker for drug response in CRC clinical therapy.

Previous reports demonstrated that HOTAIR expression is dysregulated in various types of cancers, including breast cancer [26], liver cancer [27], and lung cancer [15]. In colorectal cancer, studies have documented that HOTAIR expression in tumor tissue was higher than that in normal tissue, and upregulated HOTAIR was associated with poor clinical prognosis [28, 29]. In addition, HOTAIR is reported to contribute to drug resistance in multiple cancers [30–32]. In the present study, we found that CRC cells with high HOTAIR expression displayed high oxaliplatin resistance, and transfection of a siRNA targeting HOTAIR significantly increased the oxaliplatin sensitivity in CRC cells. In addition, sh-HOTAIR plus oxaliplatin treatment significantly reduced the weight and size of tumors compared with oxaliplatin treatment alone, suggesting that HOTAIR inhibition can reduce the resistance of CRC to oxaliplatin.

In the present study, we also found that HOTAIR inhibition significantly increased the oxaliplatin sensitivity in CRC cells and reversed EMT under hypoxia (Fig. 3). HIF-1α is the main marker of hypoxia. It has been verified that HOTAIR is regulated by HIF-1α at the transcriptional level under hypoxia [33]. Consistent with that, our study also found that HIF-1α siRNA downregulated the expression of HOTAIR and increased oxaliplatin sensitivity under hypoxic conditions (Fig. S4). However, others thought that HOTAIR acts as an upstream of HIF-1α [34, 35]. The precise regulation mechanism between HOTAIR and HIF-1α needs to be further explored.

The present study showed that HIF-1α, HOTAIR, and ZEB1 increased under hypoxia. Downregulation of HIF-1α inhibited HOTAIR/miR-1277-5p/ZEB1 and increased the chemotherapeutic sensitivity of CRC cells under hypoxia (Fig. S4). ZEB1, like other EMT transcription factors, can also bind to the promoter region of E-cadherin and inhibit its transcription and expression [36]. HIF-1α has been reported to promote EMT by regulating EMT-related genes [37, 38]. HIF-1α can inhibit ZEB1 from binding to the promoter region of ZEB1 through hypoxia response elements [36]. The recent publication demonstrates that hypoxia-induced ZEB1 is involved in EGFR inhibitor resistance in lung cancer [39]. Similarly, we found that HIF-1α siRNA inhibited the expression of ZEB1.

LncRNAs can act as a competitive endogenous RNA (ceRNA) of miRNA to regulate the target genes. Wei et al. reported that HOTAIR affected gastric cancer cell growth and metastasis via sponging miR-1277 which suppresses COL5A1 transcription [40]. Liu et al. reported that HOTAIR functions as a competing endogenous RNA to regulate HER2 expression by sponging miR-331-3p in gastric cancer [41]. Here, the database prediction identified that HOTAIR could bind to specific sites of miR-1277-5p to regulate the target ZEB1 through competitive endogenous RNA function. Further experimental verification indicated that miR-1277-5p mimics promote oxaliplatin sensitivity while miR-1277-5p inhibitor contributes to oxaliplatin resistance, indicating that miR-1277-5p acts as a tumor suppressor, which is consistent with the previous study in gastric cancer [40]. Moreover, inhibition of miR-1277-5p could reverse the effect of HOTAIR knockdown on oxaliplatin sensitivity. Hence, our research demonstrated that HOTAIR regulated oxaliplatin resistance of CRC by sponging miR-1277-5p, which clarifies the mechanism by which HOTAIR mediates hypoxia-induced oxaliplatin resistance.

In conclusion, the present study revealed a lncRNA HOTAIR/miR-1277-5p/ZEB1 axis underlying the hypoxia-mediated oxaliplatin resistance in CRC cells. Our results suggest that HOTAIR could be considered as a novel potential target to attenuate chemoresistance in CRC patients.

## Material and methods

### Cell lines and chemical reagents

Three human CRC cell lines, LoVo, HT-29, and HCT116, were purchased from the American Type Culture Collection (Manassas, VA, USA) and the Shanghai Institute of Cell Biology (Shanghai, China). LoVo and HT-29 cells were cultured in Roswell Park Memorial Institute (RPMI) 640 medium and HCT116 cells were cultured in McCOY’s 5A medium in a humidified incubator with 5% CO_2_ at 37 °C. Furthermore, the hypoxic condition in a hypoxic incubator is 5% CO2, 94% N2 and 1% O2 at 37 °C. All of the medium was supplemented with 10% fetal bovine serum (FBS).

Oxaliplatin, and antibodies recognizing ZEB1, E-cadherin, and Vimentin were purchased from Abcam (Cambridge, MA, USA). Antibodies recognizing glyceraldehyde-3-phosphate dehydrogenase (GAPDH), and horseradish peroxidase (HRP)-Goat anti-Rabbit and HRP-Goat anti-Mouse secondary antibodies were from Cell Signaling Technology (Beverly, MA, USA). Other reagents include the Click-iTEdU Imaging Kit (Invitrogen, Carlsbad, CA, USA), 2-(4-amidinophenyl)-1H-indole-6-carboxamidine (DAPI) (Sigma, St. Louis, MO, USA), and a BCA protein assay kit (Thermo Fisher Scientific, Rockford, IL, USA).

### Small Interfering RNA (siRNA) transfection

siRNAs targeting *HOTAIR* or *ZEB1* and scrambled control (GenePharma, Shanghai, China) were prepared separately in DEPC water at 50 nM and then mixed with Lipofectamine 2000 transfection reagent (Invitrogen). After incubating for 5 min at room temperature, the mixtures were added into six-well plates containing monolayers of CRC cells at 20–30% confluence and supplemented with 1 ml of OPTI-MEM (optimized minimal essential medium). After culturing for 6 h, the OPTI-MEM was changed with RPMI1640 or McCOY’s 5 A medium with 10% FBS. The sequences of siRNA targeting HOTAIR and ZEB1 were:

*HOTAIR*, si-HOTAIR_001, 5′-GAACGGGAGTACAGAGAGA-3′

si-HOTAIR_002, 5′-CCACATGAACGCCCAGAGA-3′

si-HOTAIR_003, 5′-ACCGGCGCCTTCCTTATAA-3′

*ZEB1*, si-ZEB1_001, 5′-GGCAAGTGTTGGAGAATAA-3′

si-ZEB1_002, 5′-CCAGAAATACACAGGGTTA-3′

si-ZEB1_003, 5′-GGACAGCACAGTAAATCTA-3′

### Cell proliferation assay

After receiving siRNA transfection or PBS (phosphate-buffered saline) treatment, LoVo, HT-29, and HCT116 cells were plated at 5000 cells/well in 100 µl of the corresponding medium supplemented with 0.1% FBS for 24 h. Subsequently, oxaliplatin (range: 0–10 µM) was used to treat the above CRC cells. After incubating for 48 h, 10 µl of cell counting kit-8 (CCK-8, Dojindo Laboratories, Kumamoto, Japan) reagent was added to the cells in a 96-well plate following the manufacturer’s instructions, and the absorbance at 450 nm was measured using an MRX II microplate reader (Dynex, Chantilly, VA, USA) after 2 h of incubation.

The proliferative ability of CRC cells was detected using 5-ethynyl-2´-deoxyuridine (EdU) staining in the presence of oxaliplatin. Briefly, EdU was prepared in a corresponding medium at 50 µM and added to the well to determine DNA *de novo* synthesis. The cell nuclei were marked with DAPI. The EdU stained cells were recorded under a confocal microscope.

### Western blotting analysis

Total proteins from CRC cells were extracted with Radioimmunoprecipitation assay (RIPA) buffer containing a protease inhibitor cocktail according to the manufacturer’s instructions. Protein concentrations were calculated using a BCA protein assay kit. A total of 40 µg of protein was denatured by immersion in a water bath of 95 °C for 10 min and then separated using a 10% SDS-PAGE gel. Subsequently, the proteins on the gel were electrophoretically transferred to a 0.45-µm polyvinylidene fluoride (PVDF) membrane at 350 mA for 60 min. After blocking in Tris-buffered saline supplemented with 0.1% Tween-20 (TBST) and 5% FBS for 2 h, the membrane was incubated with primary antibodies (1:1000) overnight at 4 °C. The membrane was then washed three times with TBST and incubated with secondary antibodies (1:2000) at room temperature for 2 h. Visualization was conducted using SuperSignal West Pico Chemiluminescent Substrate (Thermo Fisher Scientific) and analyzed by Image Lab software (Bio-Rad, Hercules, CA, USA).

### Quantitative real-time reverse transcription-polymerase chain reaction (qRT-PCR)

Following the manufacturers’ instructions, total RNA was extracted from CRC cells using a TRIzol RNA extraction kit (Thermo Fisher Scientific), and reverse transcribed into complementary DNA using a ReverTra Ace-α-® reverse transcription kit (Toyobo, Osaka, Japan). The qPCR was performed using the Roche LightCycler system (Roche Molecular Systems Inc., Branchburg, NJ, USA) using a Takara SYBR Premix Extaq kit (Takara, Shiga, Japan). Primers were synthesized by Shanghai Sangon Biological Engineering Technology Services Co., Ltd. (Shanghai, China) with the following nucleotide sequences:

*HOTAIR*: HOTAIR-Homo-F, 5′-AACCACGCAGAGAAATGCAG-3′

HOTAIR-Homo-R, 5′-CTCTCTGTACTCCCGTTCCC-3′

ATCB-F, 5′-TGGCACCCAGCACAATGAA-3′

ATCB-R, 5′-CTAAGTCATAGTCCGCCTAGAAGCA-3′

miR-1277, 5′-AAAUAUAUAUAUAUAUGUACGUAU-3′

### TUNEL

TUNEL was performed to detect apoptotic cells in mouse tissue slides using a TUNEL kit according to the manufacturer’s instructions (Thermo Fisher Scientific).

### Immunohistochemistry

Immunohistochemistry was performed to detect Ki-67 expression in the mouse tumor tissues. The antibodies against Ki-67was from Abcam, and the peroxidase-conjugated goat anti-rabbit immunoglobulin G (IgG) and diaminobenzidine (DAB) were from ZSGB-BIO (Beijing, China).

### Tumor xenograft experiment

All experimental protocols were approved by the Medical Ethics Committee of the First Affiliated Hospital of Zhejiang University. The experimental procedures conformed to the National Institutes of Health Guide for Care and Use of Laboratory Animals (NIH Publications, No. 8023, revised 1978). First, 1 × 10^6^ HT-29 cells were injected subcutaneously in the lateral flank of 6‐week‐old BALB/c nude mice to form tumors. Subsequently, the tumor was sectioned at 1 mm^3^ and then embedded in subcutaneous tissues. The mice were divided into four groups (*n* = 6 mice per group): control, oxaliplatin, NC plus oxaliplatin, shRNA HOTAIR plus oxaliplatin. Then, 50 µl of shRNA *HOTAIR* lentivirus at 1e^8^/ml was injected into the tumor at the appropriate volume on day 1 and day 8. For chemotherapy, oxaliplatin was injected into the tail vein at 8.3 mg/kg. The tumor volume was measured every other day and calculated following the equation: Tumor size = (Width^2^ × Length)/2.

### Statistical analysis

All experiments were conducted three times, and the data are shown as the mean ± SD. The student’s *t*-test was used to assess differences between the different groups. One-way ANOVA was used to assess differences among multiple groups. All statistical analyses were performed using SPSS (Statistical Package for the Social Sciences) 16.0 (IBM Corp., Armonk, NY, USA), and statistical significance was considered at *P* < 0.05.

## Supplementary information

supplementary file

Original Data

reply to change of authorship request

## Data Availability

All data generated or analyzed during this study are included in this published article.
