# Supplementary material for: HOTAIR/miR-1277-5p/ZEB1 axis mediates hypoxia-induced oxaliplatin resistance via regulating epithelial-mesenchymal transition in colorectal cancer
Source: Cell Death Discov. 2022 Jul 7;8:310. doi: 10.1038/s41420-022-01096-0 (PMC9263107; doi:10.1038/s41420-022-01096-0)
Supplement: Supplementary file 1 — supplementary file [file 41420_2022_1096_MOESM1_ESM.docx]

**Supplementary figures and figure legends**

**
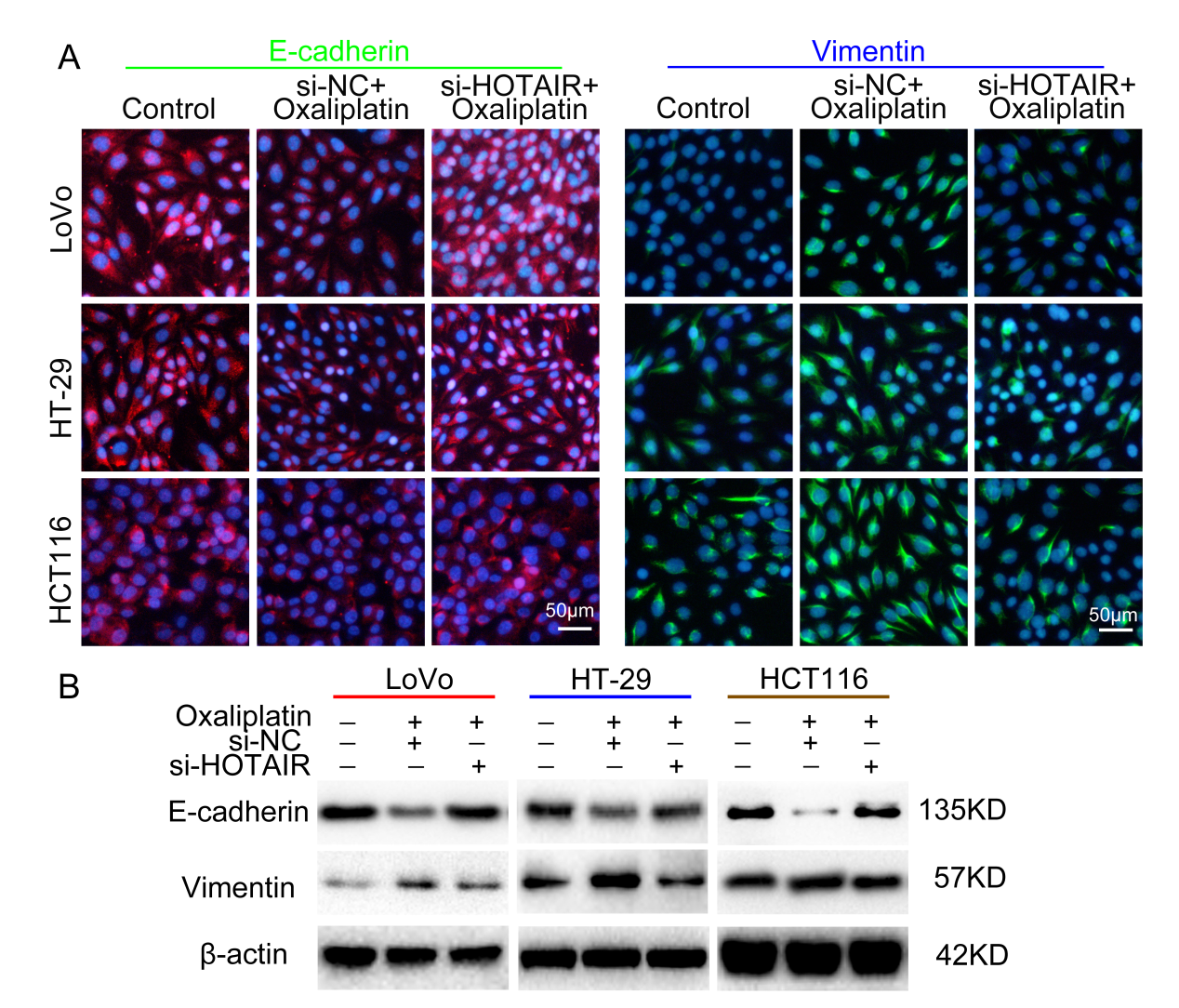
**

**Figure S1. Knockdown of HOTAIR reversed the EMT phenotype induced by oxaliplatin.**

(A-B) Confocal microscopy and western blotting assays were used to detect the E-cadherin and Vimentin expression.


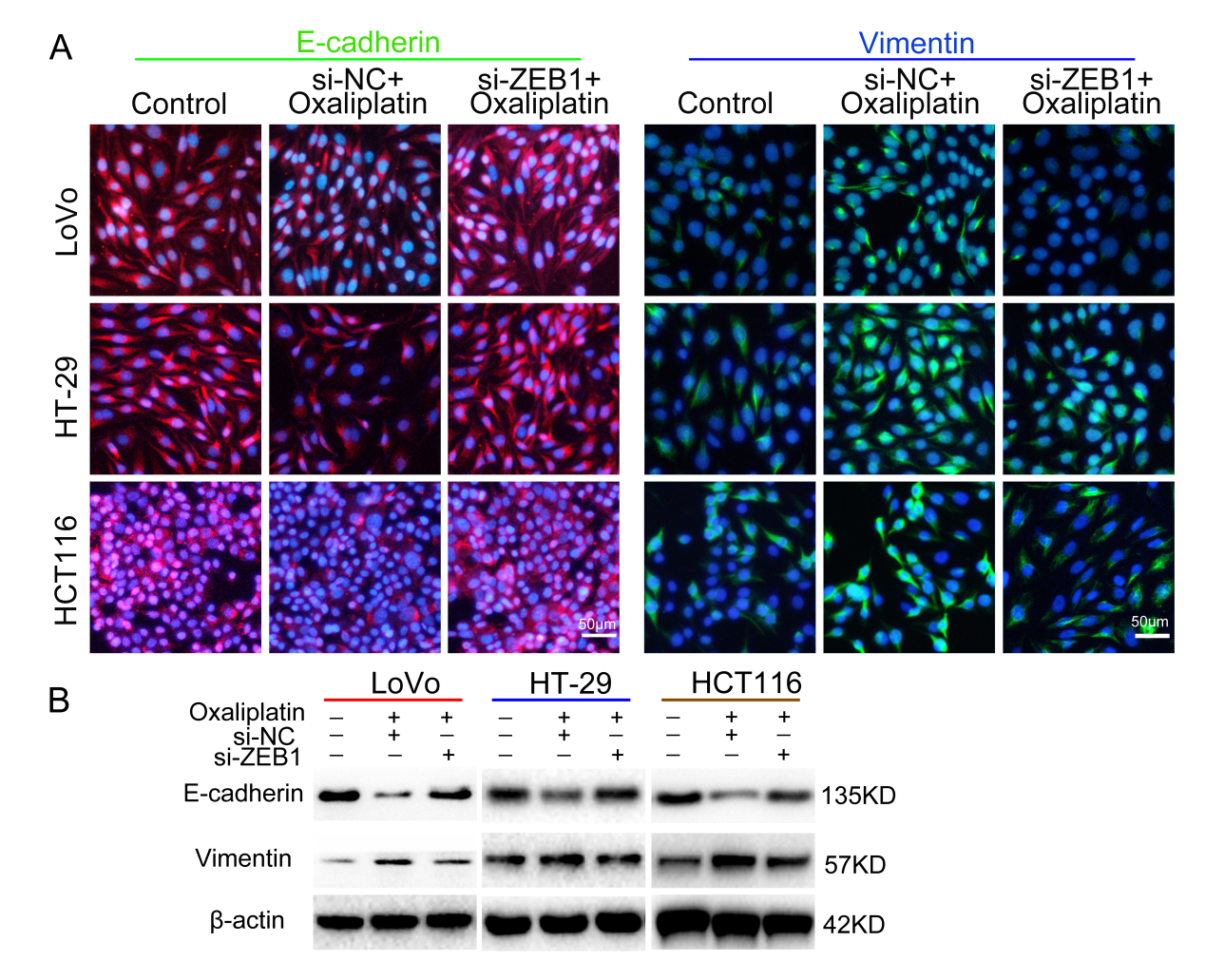


**Figure S2. Knockdown of ZEB1 reversed the EMT phenotype induced by oxaliplatin**

(A-B) Confocal microscopy and western blotting assays were used to detect the E-cadherin and Vimentin expression.


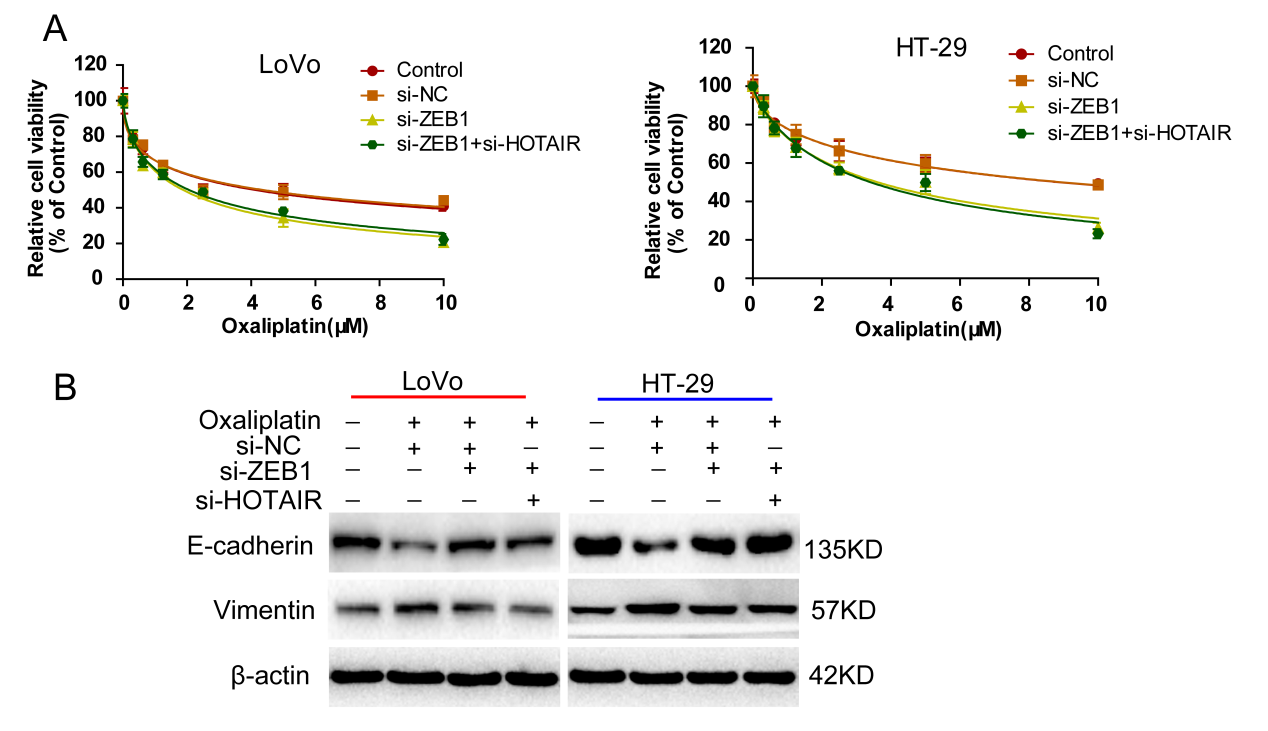


**Figure S3. Effect of ZEB1 knockdown on HOTAIR functions**

(A) The relative cell viability of CRC cells was detected by CCK-8 assay. (B) Western blotting assay was used to detect the E-cadherin and Vimentin expression.


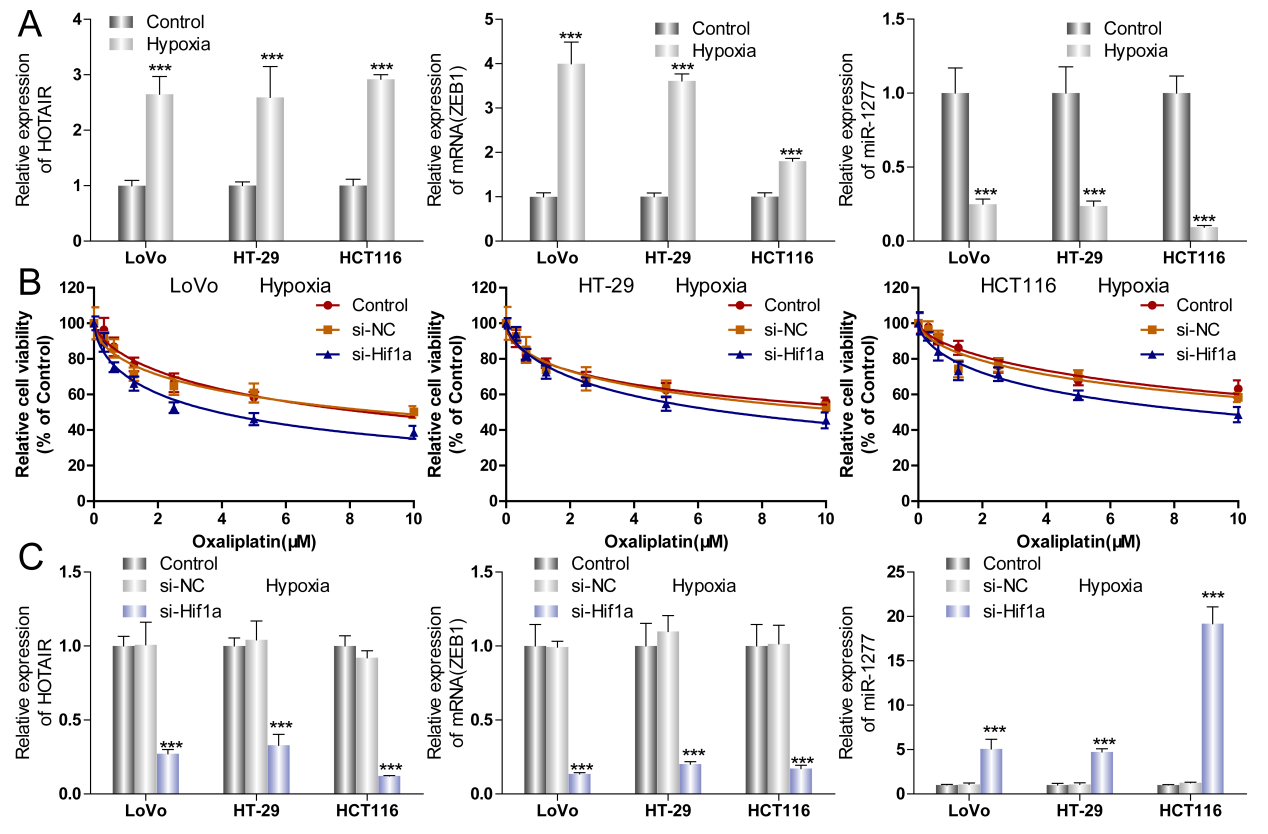


**Figure S4. Effect of hypoxia knockdown on HOTAIR, miR-1277-5p, and ZEB1 expression**

(A) qRT-PCR was used to detect the HOTAIR, miR-1277-5p, and ZEB1 expression under hypoxia. (B) The relative cell viability of CRC cells was detected by CCK-8 assay. (C) qRT-PCR was used to detect the HOTAIR, miR-1277-5p, and ZEB1 expression after si- HIF-1α treatment.
