# Supplementary material for: HOTAIR/miR-1277-5p/ZEB1 axis mediates hypoxia-induced oxaliplatin resistance via regulating epithelial-mesenchymal transition in colorectal cancer
Source: Cell Death Discov. 2022 Jul 7;8:310. doi: 10.1038/s41420-022-01096-0 (PMC9263107; doi:10.1038/s41420-022-01096-0)

Original Data

Figure 1

Hif1α:
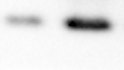

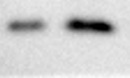

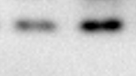


β-actin:
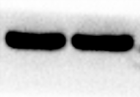

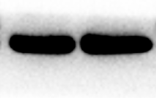

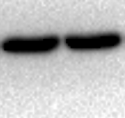


Figure 3

E-cadherin:
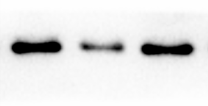

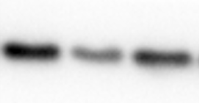


Vimentin:
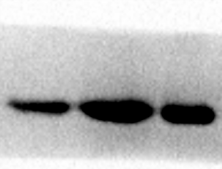

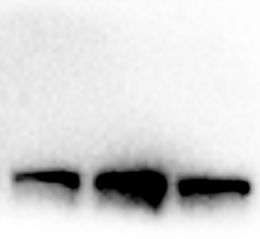


β-actin:
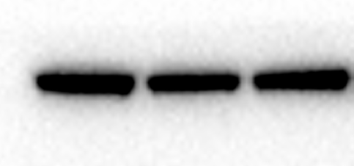

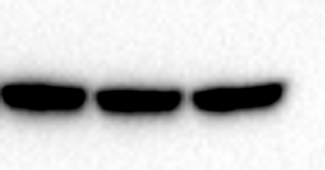


Figure 4

A ZEB1
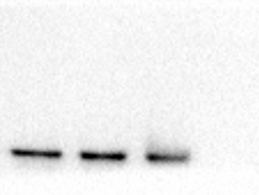

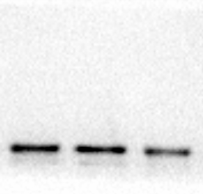

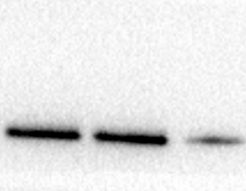


β-actin
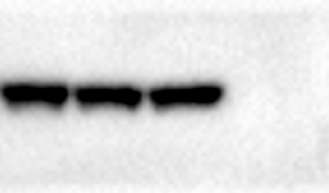

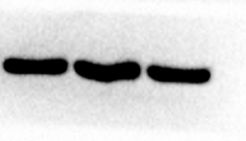

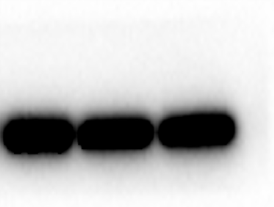


B ZEB1
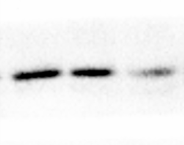

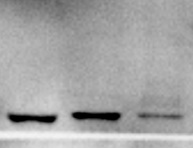

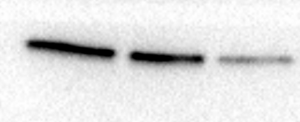


β-actin
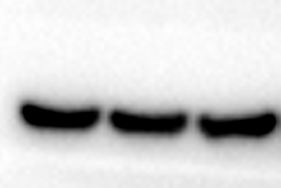

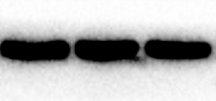

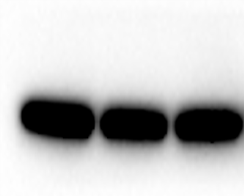

Supplement: Supplementary file 2 — Original Data [file 41420_2022_1096_MOESM2_ESM.docx]
