# Supplementary figures and images for: HOTAIR/miR-1277-5p/ZEB1 axis mediates hypoxia-induced oxaliplatin resistance via regulating epithelial-mesenchymal transition in colorectal cancer
Source: Cell Death Discov. 2022 Jul 7;8:310. doi: 10.1038/s41420-022-01096-0 (PMC9263107; doi:10.1038/s41420-022-01096-0)

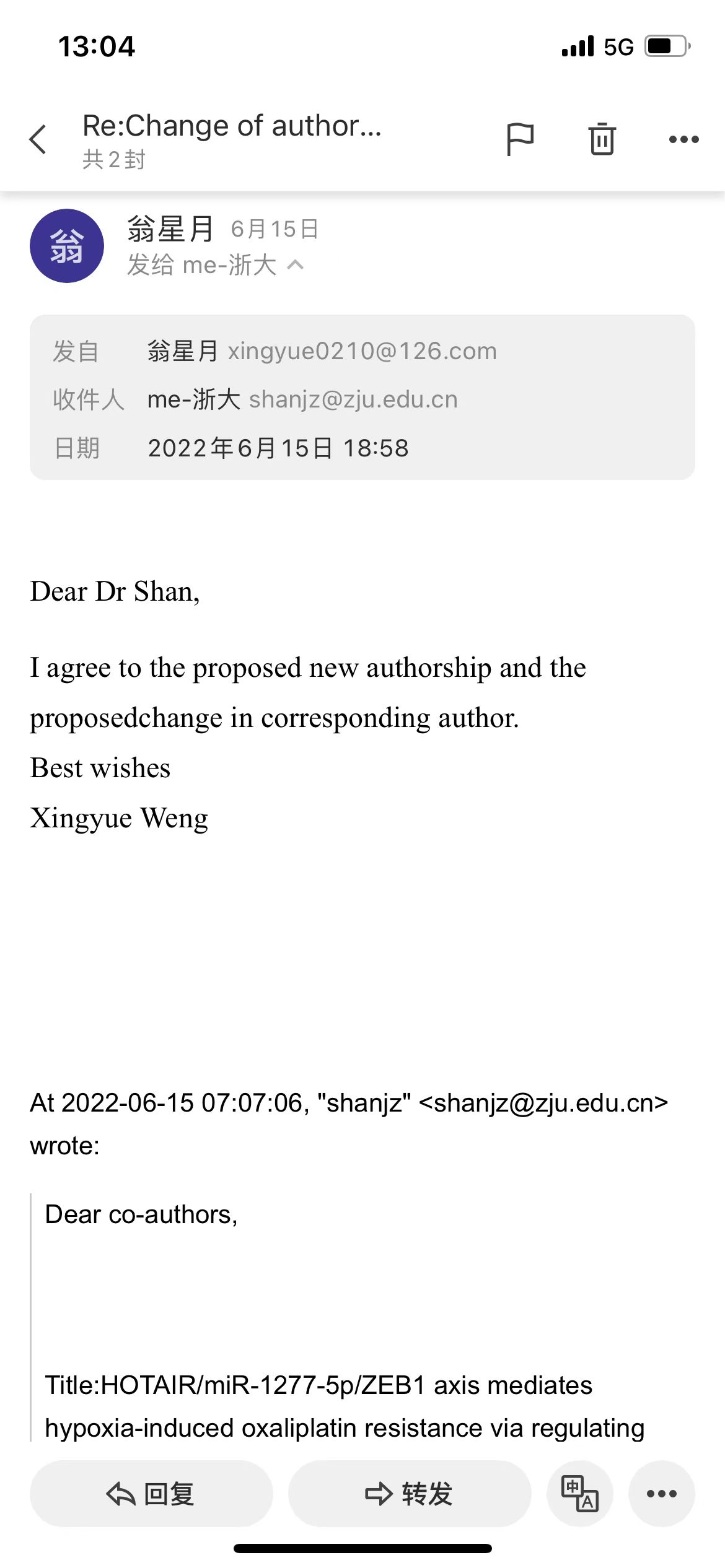

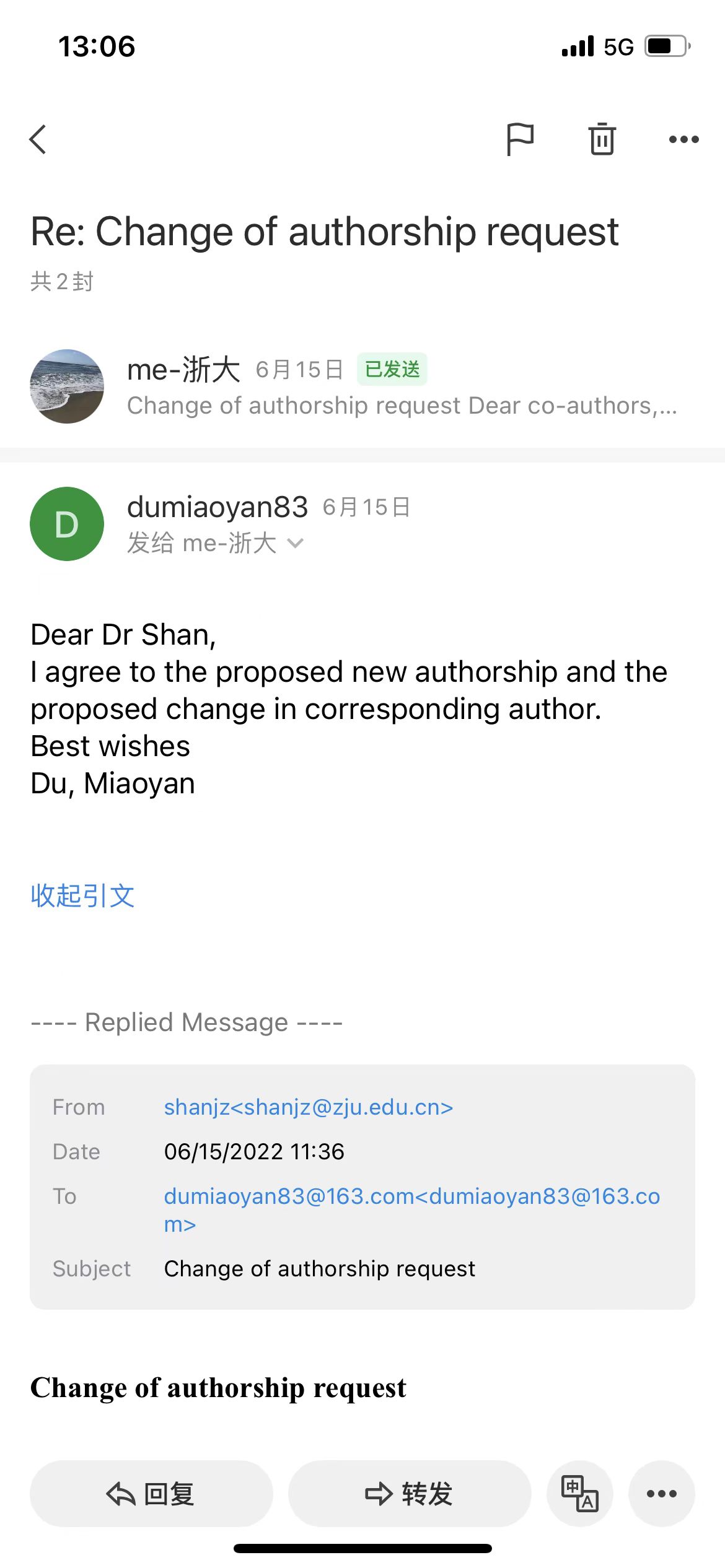


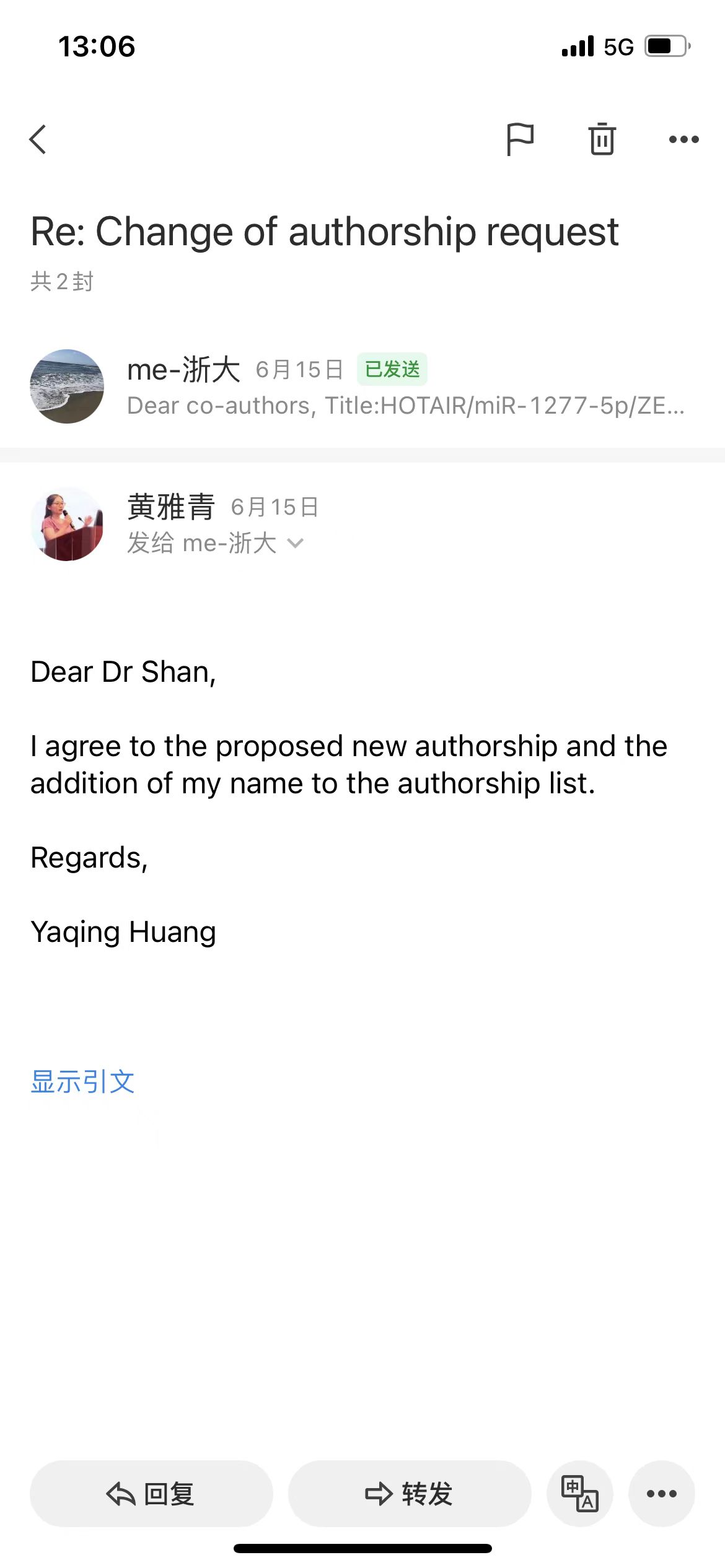


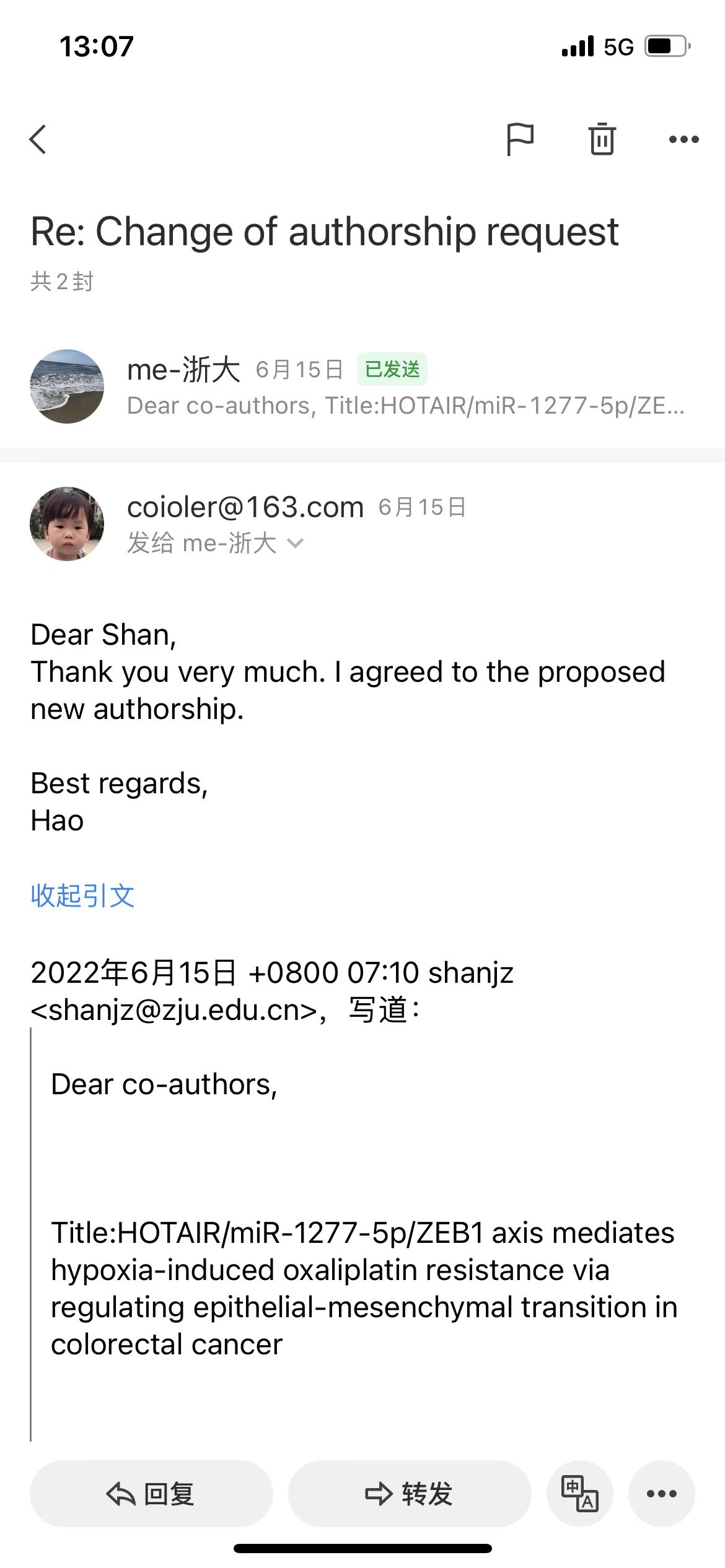


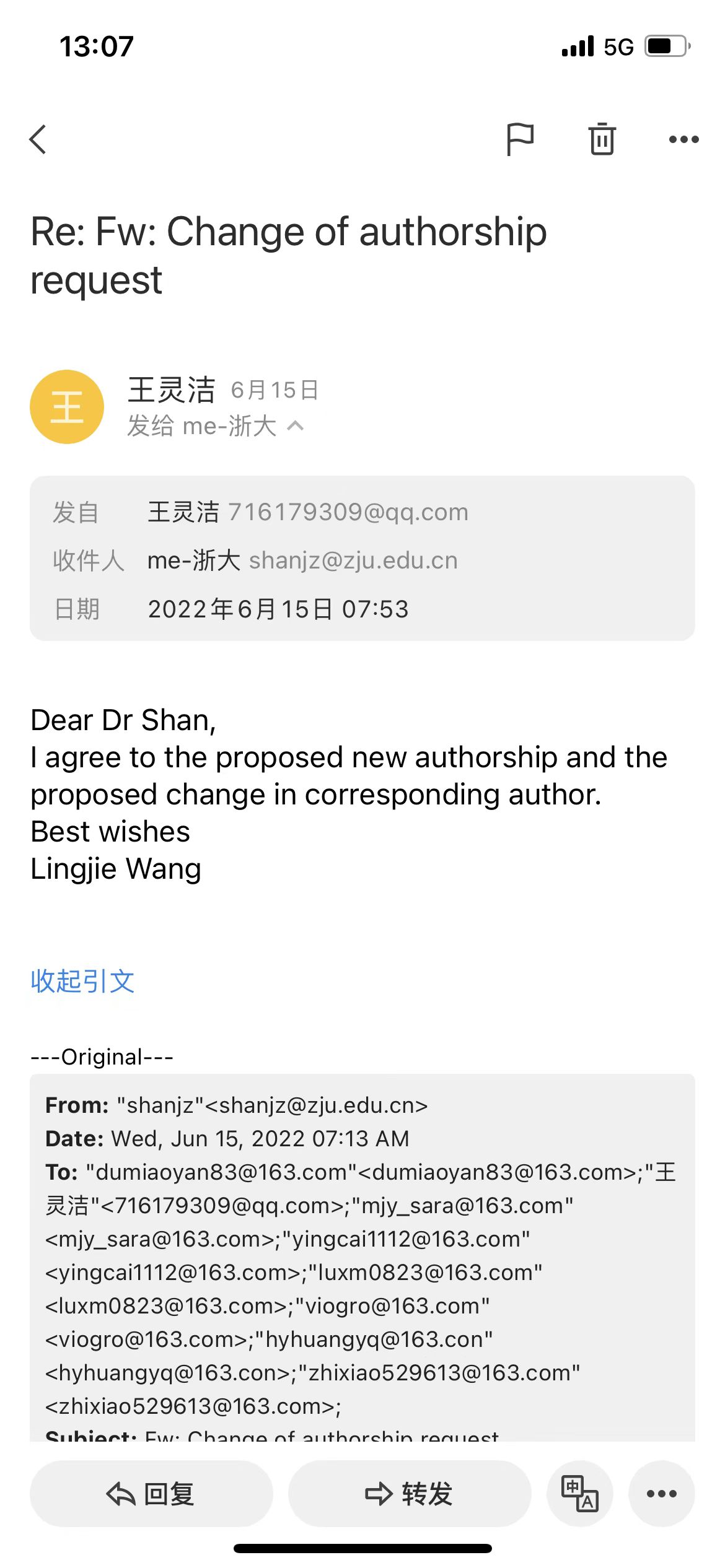


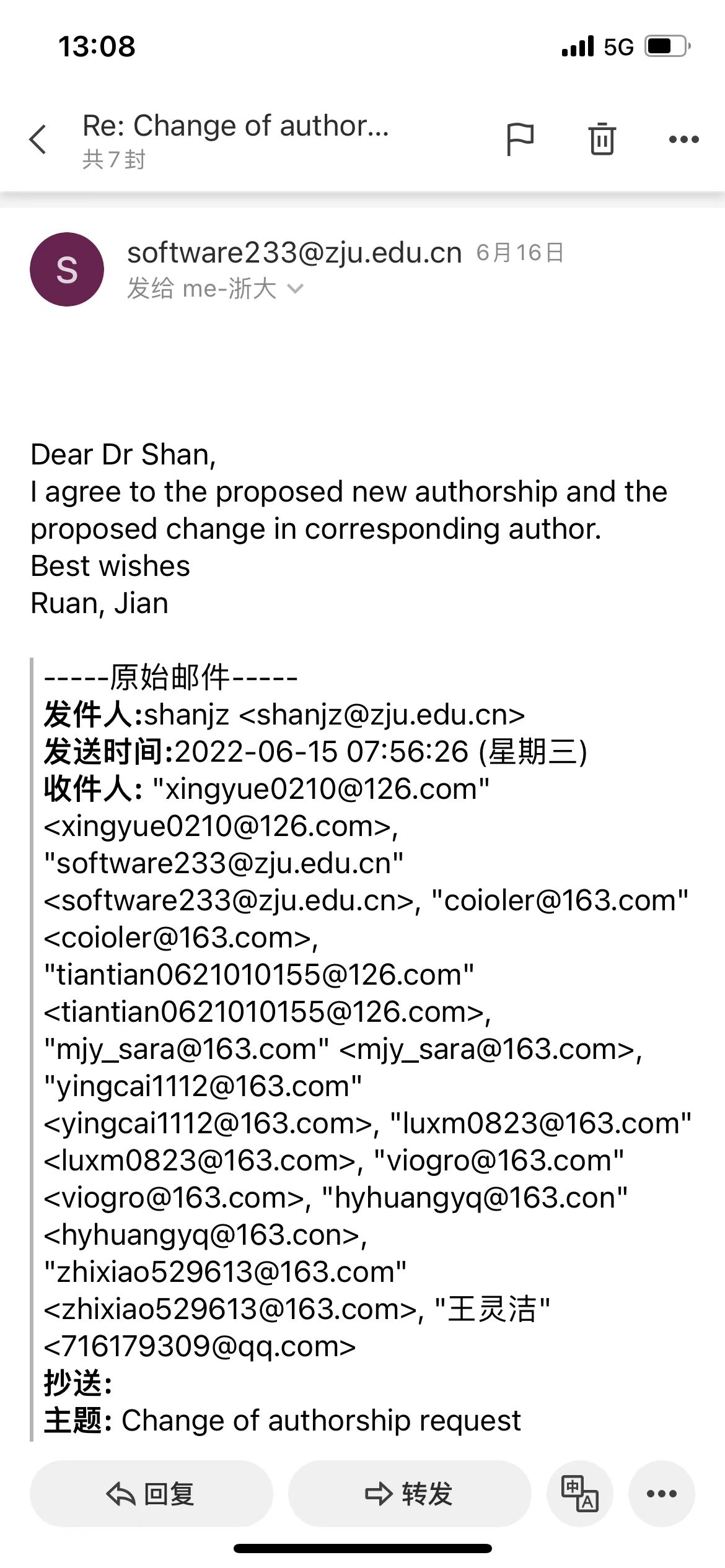


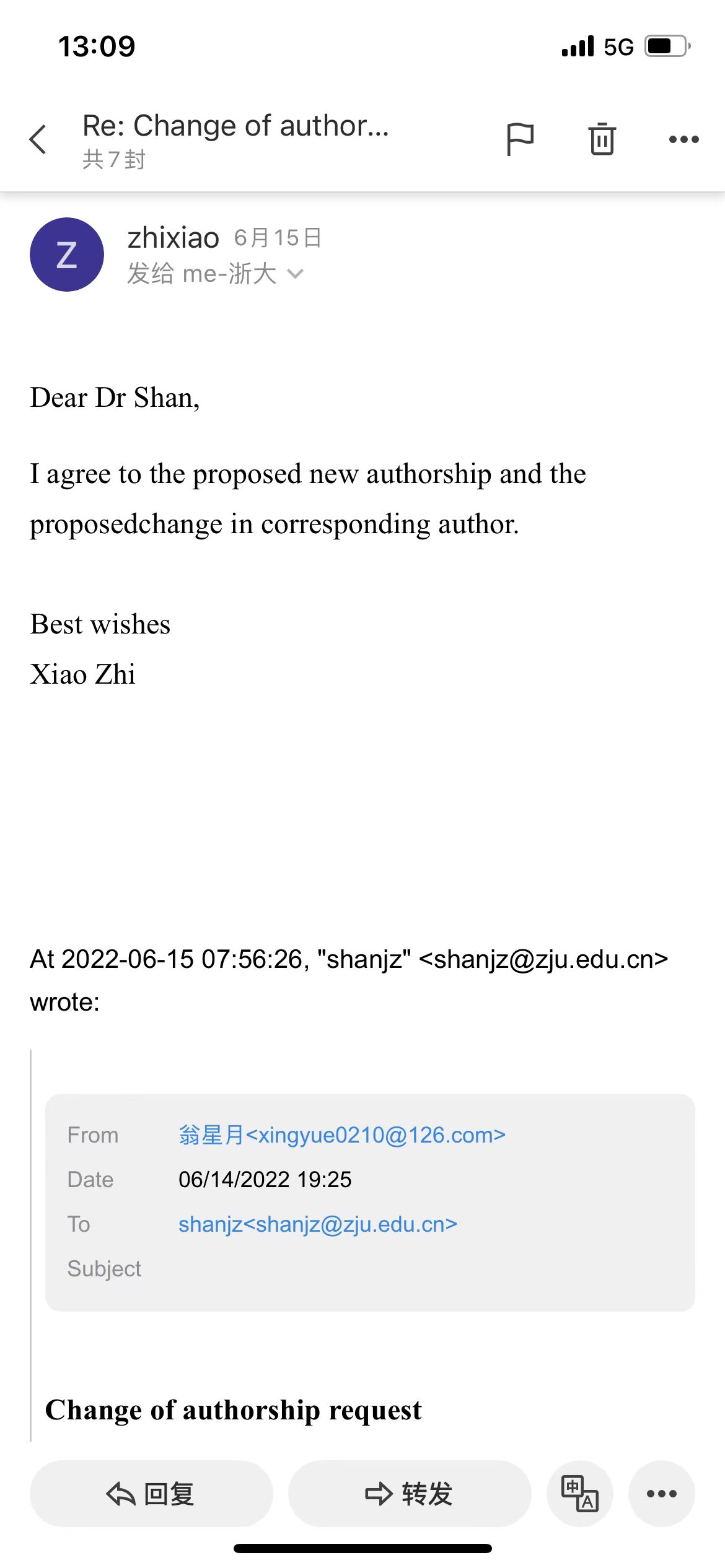


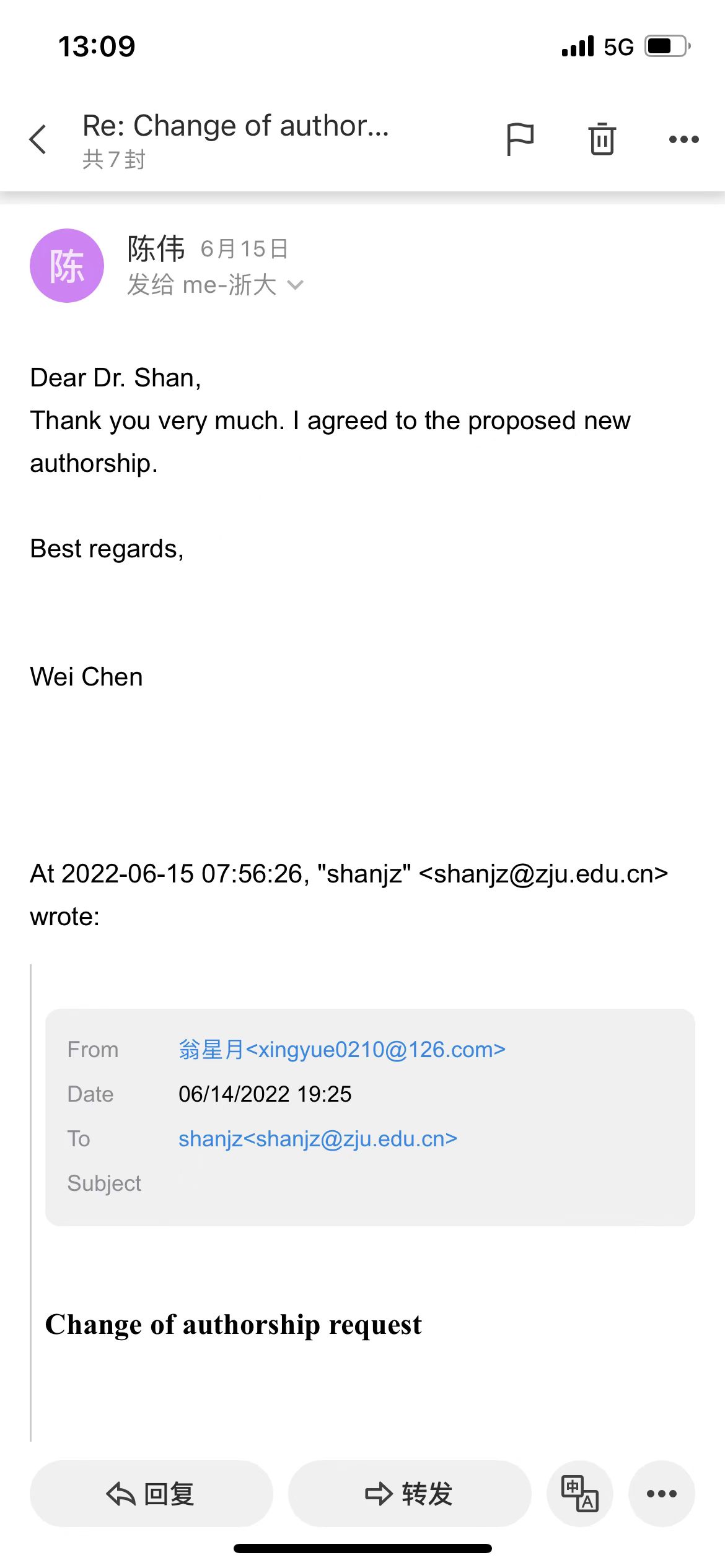


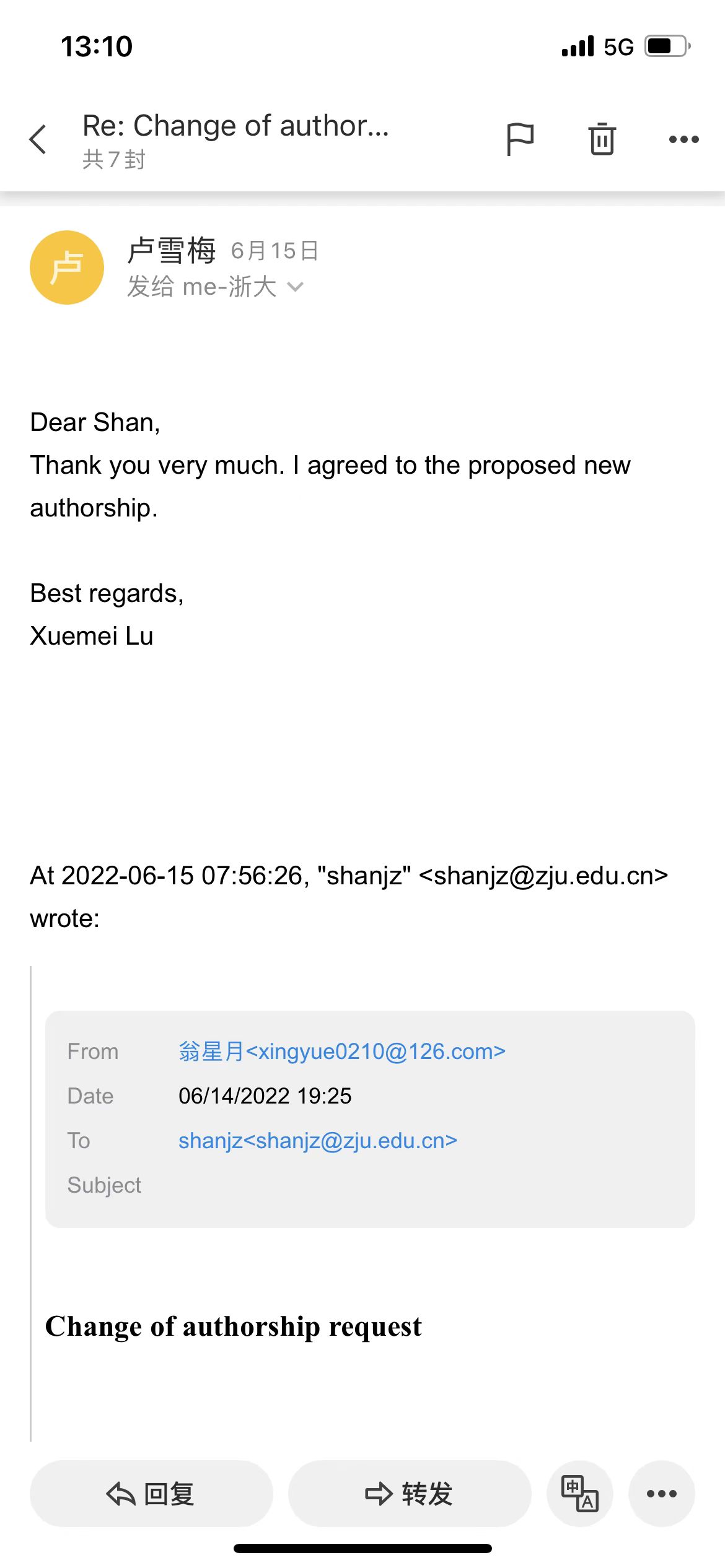


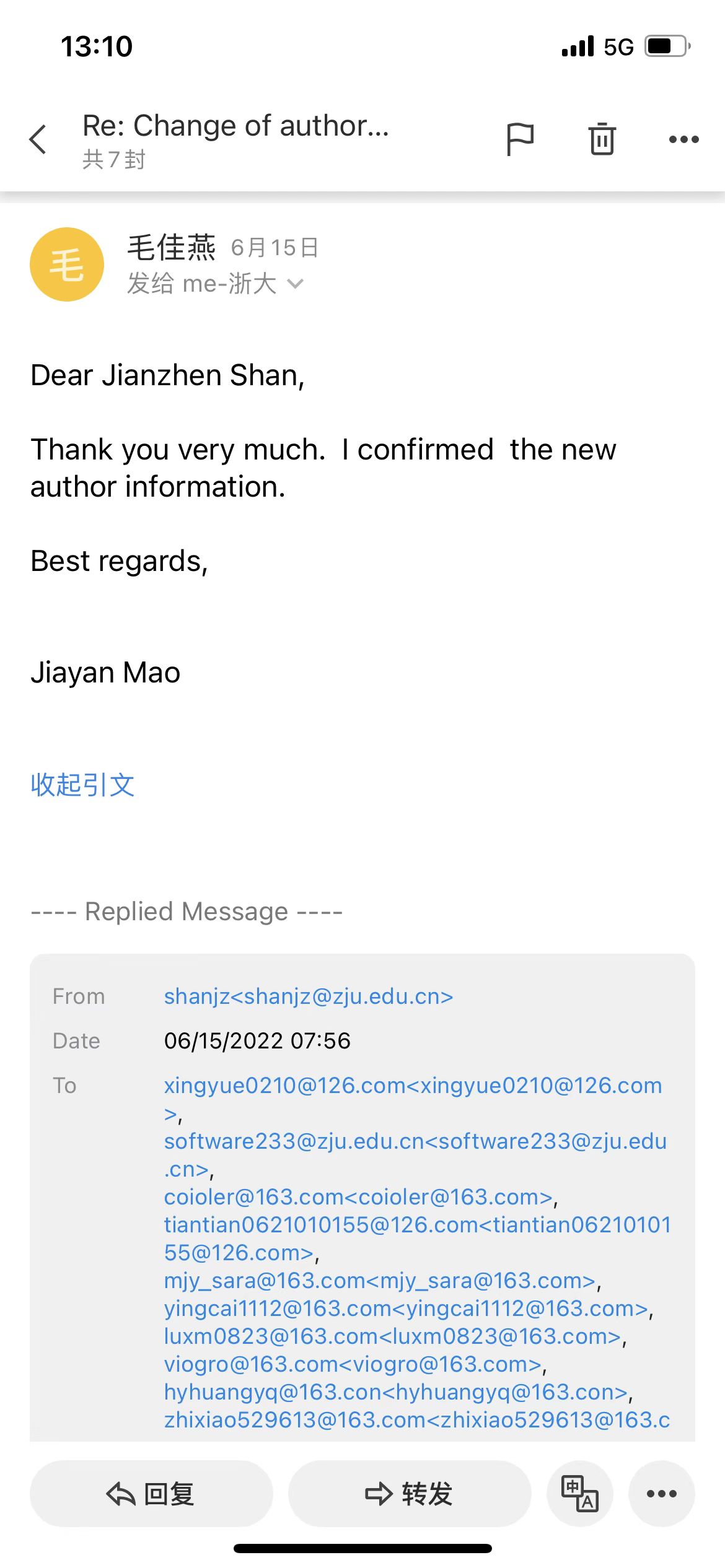


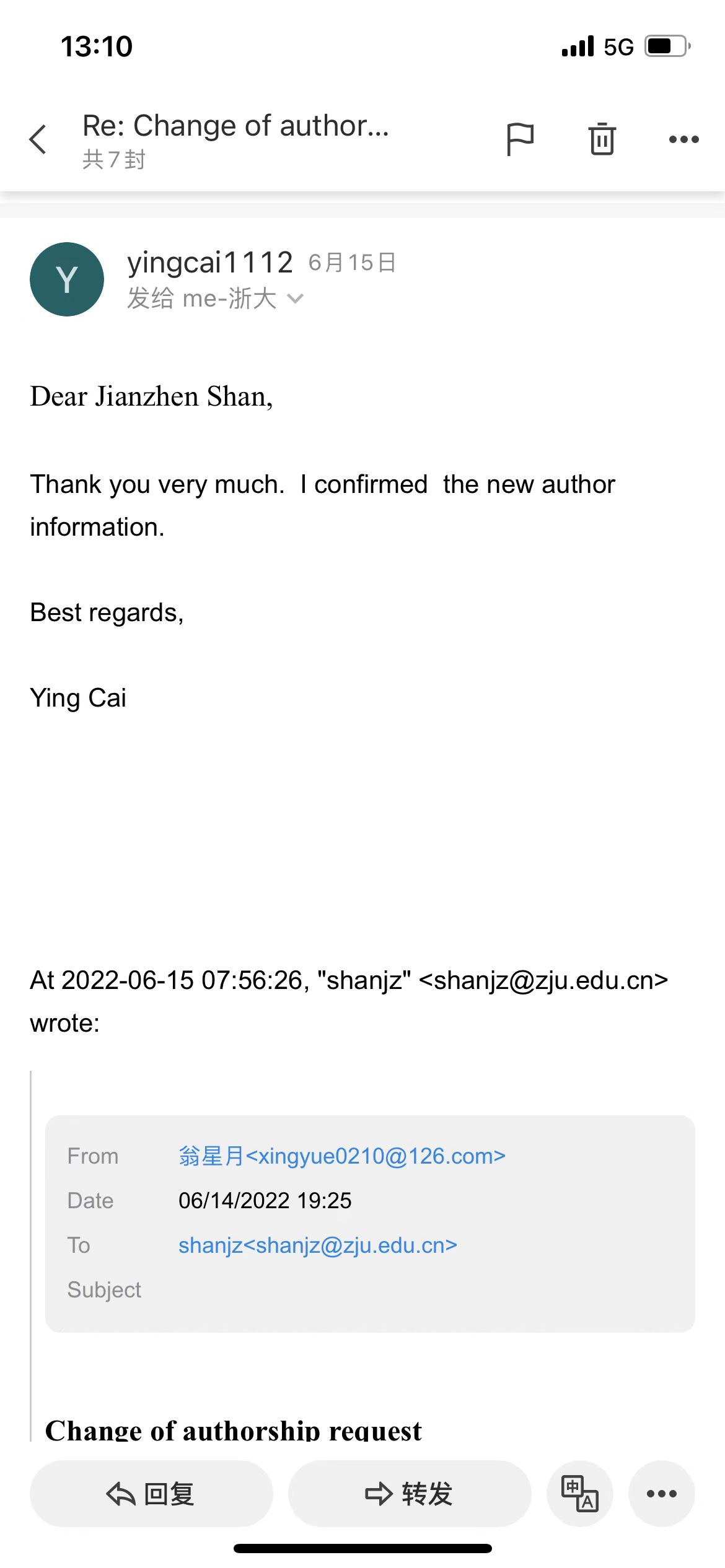

Supplement: Supplementary file 3 — reply to change of authorship request [file 41420_2022_1096_MOESM3_ESM.docx]
